# Assessment of microbial populations within Chicago area nearshore waters and interfaces with river systems

**DOI:** 10.1016/j.dib.2015.09.004

**Published:** 2015-09-25

**Authors:** Emily Sible, Alexandria Cooper, Kema Malki, Katherine Bruder, Thomas Hatzopoulos, Siobhan C. Watkins, Catherine Putonti

**Affiliations:** a Department of Biology, Loyola University Chicago, 1032W Sheridan Rd, Chicago, IL 60660, USA; b Bioinformatics Program, Loyola University Chicago, 1032W Sheridan Rd, Chicago, IL 60660, USA; c Department of Computer Science, Loyola University Chicago, 820N Michigan Ave, Chicago, IL 60611, USA

**Keywords:** Microbial community, Metagenomics, Freshwater, Lake Michigan

## Abstract

The Chicago area locks separate and control water flow between the freshwaters of Lake Michigan and the network of Illinois waterways. Under extreme storm conditions, however, the locks are opened and storm waters, untreated waste, and runoff are released directly into the lake. These combined sewer overflow (CSO) events introduce microbes, viruses, and nutrients such as nitrogen and phosphorous into nearshore waters which likely affect the native species. We collected surface water samples from four Chicago area beaches – Gillson Park, Montrose Beach, 57th Street Beach, and Calumet Beach – every two weeks from May 13 through August 5, 2014. Sampling was conducted with four biological replicates for each sampling date and location, resulting in 112 samples. Each community was surveyed through targeted sequencing of the V4 16S rRNA gene. Technical replicates were also sequenced and are included in this dataset. Taxa were identified using Mothur. Raw sequence data is available via NCBI׳s SRA database (part of BioProject PRJNA245802).

## **Specifications table**

**Table:** 

Subject area	*Biology*
More specific subject area	*Bacterial metagenomics*
Type of data	*Text files: sequences*
How data was acquired	*Illumina MiSeq Desktop Sequencer*
Data format	*Raw*
Experimental factors	*DNA extracted from bacterial cells captured using 0.22* *μm filters.*
Experimental features	*Amplification of the V4 region of the 16S rRNA gene. Sequencing using the MiSeq Reagent Kit v2 (500-cycles) kit for the Illumina MiSeq platform.*
Data source location	Chicago, IL, USA: Montrose Beach (41°58′0.71″N, 87°38′13.35″W), 57^th^ Street Beach (41°47′25.54″N, 87°34′41.25″W), and Calumet Beach (41°43′8.18″N, 87°31′32.51″W); Wilmette, IL, USA: Gillson Park (42°4′45.10″N, 87°40′59.10″W).
Data accessibility	*Raw data is available through NCBI׳s BioSample database by following this link. BioSample IDs include: SAMN03106100, SAMN03408290, SAMN03417850, SAMN03431409, SAMN03431410, SAMN03431411, SAMN03431413, SAMN03431415, SAMN03431417, SAMN03431418, SAMN03431419, SAMN03431420, SAMN03431421, SAMN03431422, SAMN03431423, SAMN03431424, SAMN03431425, SAMN03431426, SAMN03431427, SAMN03431428, SAMN03431429, SAMN03431430, SAMN03431431, SAMN03431432, SAMN03431433, SAMN03431434, SAMN03431435, SAMN03431436, and SAMN03431437.*

## **Value of the data**

• This dataset includes microbial surveys (with replication) including an instance in which the Chicago lock system was open, releasing rain, sewage water, and runoff into the nearshore waters and thus disturbing the native microbial communities.
• The raw metagenome data is publicly available for further analysis and comparison to microbial communities within other urban and rural freshwater environments.
• The sampling regime provides the opportunity to consider temporal and spatial variation between microbial communities within the nearshore waters, particularly in comparison with our laboratory*׳s* prior sequencing efforts during 2013.

## 1 Experimental design, materials and methods

### 1.1 Sample collection

Four Chicago area beaches were selected as study sites: Gillson Park (42°4′45.10″N, 87°40′59.10″W), Montrose Beach (41°58′0.71″N, 87°38′13.35″W), 57th Street Beach (41°47′25.54″N, 87°34′41.25″W), and Calumet Beach (41°43′8.18″N, 87°31′32.51″W). All four are recreational swimming areas. The Montrose Beach sampling site is bordered to the north by the Montrose Beach dog park and to the south by the Montrose Harbor Marina. 57th Street Beach and Calumet Beach are used solely for swimming. Gillson Park is located north of Chicago in Wilmette, IL; this beach is also recreational and adjacent to the north of Wilmette Harbor. Gillson Park and Calumet Beach are adjacent to locks controlling the movement of water between the North Shore Channel and Calumet River, respectively, and Lake Michigan. (No specific permits or permissions were required for the water samples collected from the Chicago nearshore waters; a permit was obtained for Gillson Park in accordance with the Wilmette Park District.) Each site was sampled with four replicates every two weeks over the three month period – May 13 through August 5, 2014. Water was collected from the surface at a distance from the shore such that the water level was approximately knee-deep (~0.5 m deep). Each sample (4 L), including each biological replicate, was collected within a 5 m area.

### 1.2 Bacterial Isolation

Isolation of bacterial cells was conducted through filtration. The water was first filtered through sterile 0.45 μm bottle-top cellulose acetate membrane filters (Corning Inc, Corning, NY) to remove plant matter, sand, debris, and eukaryotic cells. The filtrate was then passed through a 0.22 μm polyethersulfone membrane filter (MO BIO Laboratories, Carlsbad, CA) to capture bacterial cells. Each 4 L sample was passed through a single filter. The filters were then stored at −20 °C until extraction.

### 1.3 DNA extraction

DNA was extracted using the MO BIO Laboratories PowerWater® DNA Isolation Kit (Carlsbad, CA). The protocol recommended by the manufacturer was followed with the exception of an additional heat treatment at 65 °C for 10 min prior to initial vortexing. DNA isolated from each of the individual samples for a given collection date/location was pooled together. Concentrations were verified using the Qubit® Fluorometer (Life Technologies, Carlsbad, CA). DNA was stored at −20 °C until sequencing.

### 1.4 16S rRNA Amplification

The V4 region of the 16S rRNA sequence was amplified using the primer combination of 5′-TCG TCG GCA GCG TCA GAT GTG TAT AAG AGA CAG GTG CCA GCM GCC GCG GTA A-3′ (forward) and 5′-GTC TCG TGG GCT CGG AGA TGT GTA TAA GAG ACA GGG ACT ACH VGG GTW TCT AAT-3′ (reverse) (Integrated DNA Technologies, Coralville, IA). These primers include the Illumina adapter overhang nucleotide sequences as well as V4-specific sequences producing an amplicon ~359 bp in length. This initial PCR reaction was performed as follows: 2 μL of each primer (200 ng/μL), 8 μL DNTPs (Promega, Madison, WI) at a 1.25 mmolar/nucleotide concentration, 1 μL of bacterial DNA, 28.5 μL of nuclease free water and the Platinum® Taq (Life Technologies, Carlsbad, CA) components of DNA polymerase (0.5 μL), 10× PCR R×n Buffer (5 μL), and 50 mM MgCl_2_ (3 μL). Each reaction was amplified as follows: initial denaturing at 94 °C for 2 min, 30 cycles of 94 °C for 30 s, 55 °C for 30 s, and 72 °C for 1 min, followed by a final extension at 72 °C for 7 min. Amplification was verified via gel electrophoresis in a 1% agarose gel. Negative controls were also run to confirm there was no contamination within the samples as a residual of the reagents or extraction protocol.

### 1.5 Index PCR

To facilitate multiplexing, each PCR product was subsequently amplified again using primers including the Illumina adapter sequences and indexing sequences for subsequent de-multiplexing. Samples were multiplexed using the NEBNext® Multiplex Oligos for Illumina® (Dual Index Primers Set 1) (New England Biolabs, Ipswich, MA). Subsequent DNA preparation – PCR clean-up, library pooling, and sample loading – followed the standard protocols established by Illumina for the MiSeq Benchtop Sequencer [1]. Sequencing was performed using the Illumina MiSeq Benchtop Sequencer (Loyola University Chicago׳s Center for Biomedical Informatics, Maywood, IL). Paired end reads, each 250 nucleotides in length, were produced using the Illumina MiSeq Reagent Kit v2 (500-cycles).

### 1.6 Sequence demultiplexing

Demultiplexing of the sequence data was automated by the Illumina sequencer׳s CASAVA package.

### 1.7 Taxonomic classification

Sequence analysis was conducted using the mothur package [2] following the protocol for sequences generated by the MiSeq platform [3]. The fastq files generated were first assembled into contigs and subsequently filtered using mothur commands to remove contigs containing putative sequencing errors as well as chimeras (uchime). Reads for which the paired-ends could not be assembled were removed from further analysis. Next, the filtered reads were compared against a local copy of the Silva database [4] in order to ascertain the taxonomy of each read; a cutoff threshold (bootstrap) of 80% was used. OTU clustering was performed using mothur׳s cluster.split command, split to the level of Order (taxlevel=4). Batch files were created to streamline the analysis.
